# Surgical Therapeutics

**Published:** 1882-08

**Authors:** J. R. Haynes

**Affiliations:** Indianapolis; Bureau of Surgical Therapeutics, I. H. A.


### SURGICAL THERAPEUTICS.

J. R. HAYNES, M. D., INDIANAPOLIS.

(*Bureau of Surgical Therapeutics, I. H. A.*)

The subject of surgical therapeutics seems to me to be so plain that it requires but little said upon the subject. In all cases of operative surgery, I make use of no drugs for external appliances in any form, whatever.

In the dressing of accidental or operative surgical wounds, I endeavor to assist nature, first, after operation to carefully prepare the wound, and then to close it as accurately as possible by suture or otherwise, as the emergency of the case demands. Then carefully apply two or three layers of the roller bandage over the wound and adjacent parts, so as to keep all external irritation from it, together with the irritating influences of the atmosphere; this I allow to remain until the wound is healed, unless pus should form, or the parts become painful; in either case, remove the dressing, or so much of it as will give relief, or for the purpose of properly cleansing, and should the case require look after the proper drainage; should there be found any pus or discharge, it should be carefully wiped away, but *no* water or other irritating substance should be allowed to come in contact with any part of the wound where the skin is broken, until it has properly healed or a new skin has formed and become strong enough to withstand proper manipulating.

In lacerated wounds if the edges are jagged and torn, trim off such parts with sharp knife or scissors as you are certain will slough; and after carefully securing such bloodvessels as are necessary, cleanse and carefully close the wound so as to accurately bring the edges in exact apposition by suture, plaster, silver pins or roller, as the case demands, and then carefully protect it from all irritating, or atmospherical influences, by the application of the proper roller bandage, so as to completely cover the wound and adjacent parts, and allow *no* other application whatever. If the wound should be upon exposed parts where the roller could not be applied, such as scalp wounds, trim the hair sufficient so as to allow the proper application of the requisite number of sutures to hold the edges in proper apposition, and then, after the proper cleansing and closing the wound, apply two or three thicknesses of muslin of a sufficient size to cover the wound and adjacent parts, and contrive some plan to hold it in place so as to keep all irritating and atmospherical influences from it, and in from three to five days remove the sutures and your case is well.

In wounds upon the face or any exposed parts, never use anything but silver pins, made in the form of hair-lip pins, and apply the figure of eight (regular hair-lip) suture and carefully cover to keep out the air and keep dry. These pins may be used in the same way in any wound unless the wound is of very large size and protected from the irritating influences of the air, and do not remove any of the dressing unless pus should form and then only enough for proper drainage until they are properly healed, when you may remove all the dressings, and if carefully done there will remain *no* permanent scar.

Not long since I had the care of a little girl whose face had been fearfully torn by a vicious dog. I expected there would remain some frightful scars, but the wounds were carefully approximated and held by different sized silver pins with the hair-lip suture, and the air thoroughly excluded from the wounds; they united by first intention, *no pus* was formed, the sutures and pins were removed in five days, and three months after, no marks of the wound were visible to the naked eye.

If pure silver is used for the pins they will cause no irritation or sloughing; if the air is kept out, the parts will heal nicely and *no* pus will form, and if carefully done will leave no mark, even if they are pretty severe.

I then endeavor to select the proper simillimum to the case, the same as if there was *no* wound under consideration.

If I have been compelled to resort to an anæsthetic, in that case, if there are symptoms of great depression, give two or three doses of Opium (6) sixth, which relieves from the prostration produced by the chloroform (I never use anything as an anæsthetic but chloroform, the best I can get), and if the shock is severe following the operation, give two or three doses of Aconite 1m and do not repeat unless positively necessary.

Should traumatic fever threaten, follow with Arnica $2^2$ to 10m every two hours, until the danger is passed, and then stop all medication unless some new complication should arise—in that case select the proper simillimum.

If the nerves have been greatly injured or bruised, I would if indicated give Hypericum, or if the periosteum was lacerated I

should think of Ruta, or the bone fractured, should take Symphytum into consideration. But always endeavor to select the proper simillimum to the case in hand, and be careful *not* to repeat the dose too often or to continue it too long.

I do not think it makes *so* very much difference as to the potency of the drug given as it does to be sure you have selected the *right* one; but I believe the 10m will relieve and cure in one-fourth the time and with more certainty, than the sixth will, and all potencies below will be more tardy in their action even if the system will respond to them at all.

